# GD2 CAR T cells against human glioblastoma

**DOI:** 10.1038/s41698-021-00233-9

**Published:** 2021-10-27

**Authors:** Malvina Prapa, Chiara Chiavelli, Giulia Golinelli, Giulia Grisendi, Marco Bestagno, Rosanna Di Tinco, Massimiliano Dall’Ora, Giovanni Neri, Olivia Candini, Carlotta Spano, Tiziana Petrachi, Laura Bertoni, Gianluca Carnevale, Giuseppe Pugliese, Roberta Depenni, Alberto Feletti, Corrado Iaccarino, Giacomo Pavesi, Massimo Dominici

**Affiliations:** 1 Laboratory of Cellular Therapy, Division of Oncology, Department of Medical and Surgical Sciences for Children & Adults, University of Modena and Reggio Emilia, Modena, Italy; 2 International Centre for Genetic Engineering and Biotechnology, Trieste, Italy; 3 Department of Surgery, Medicine, Dentistry and Morphological Sciences with Interest in Transplant, Oncology and Regenerative Medicine, Modena, Italy; 4 Rigenerand Srl, Medolla, Modena, Italy; 5 Clinical and Experimental Medicine PhD Program, University of Modena and Reggio Emilia, Modena, Italy; 6 Technopole Mario Veronesi of Mirandola, Fondazione Democenter, Mirandola, Modena, Italy; 7 Department of Oncology and Hematology, University-Hospital of Modena and Reggio Emilia, Modena, Italy; 8 Department of Neurosciences, Biomedicine and Movement Sciences, Institute of Neurosurgery, University of Verona, Verona, Italy; 9 Department of Biomedical, Metabolic and Neural Sciences, University of Modena and Reggio Emilia- Division of Neurosurgery, Department of Neurosciences, University-Hospital of Modena and Reggio Emilia, Modena, Italy

**Keywords:** Cancer immunotherapy, CNS cancer

## Abstract

Glioblastoma is the most malignant primary brain tumor and is still in need of effective medical treatment. We isolated patient-derived glioblastoma cells showing high GD2 antigen expression representing a potential target for CAR T strategy. Data highlighted a robust GD2 CAR antitumor potential in 2D and 3D glioblastoma models associated with a significant and CAR T-restricted increase of selected cytokines. Interestingly, immunosuppressant TGF β1, expressed in all co-cultures, did not influence antitumor activity. The orthotopic NOD/SCID models using primary glioblastoma cells reproduced human histopathological features. Considering still-conflicting data on the delivery route for targeting brain tumors, we compared intracerebral versus intravenous CAR T injections. We report that the intracerebral route significantly increased the length of survival time in a dose-dependent manner, without any side effects. Collectively, the proposed anti-GD2 CAR can counteract human glioblastoma potentially opening a new therapeutic option for a still incurable cancer.

## Introduction

Glioblastoma is the most malignant and common variant of a wide spectrum of primary glial brain tumors. The average survival period for patients with glioblastoma is around 15 months^1^. Although gradual improvements in survival rates and quality of life for glioblastoma patients have been noted, more medical breakthroughs are required^2^. The location of the disease and the infiltrative capability of glioblastoma make complete surgical resection extremely difficult. Moreover, chemotherapy and irradiation regimens are not curative; thus, almost all patients experience tumor progression or recurrence that lead to devastating consequences^3^. Since no standard of care is established for recurrent or progressive glioblastoma^4^, the demand for new treatment modalities offering better clinical outcomes for these patients is in high demand. From this perspective, immunotherapy regimens with chimeric antigen receptor (CAR) T cells may provide a potentially effective strategy to target specific tumor antigens, thus reaching residual glioblastoma cells^5^.

CARs are recombinant receptors that redirect the specificity and the function of T lymphocytes and other immune cells to antigens expressed on the surface of tumor cells in an MHC-independent manner. Upon recognition of antigens, CAR T cells execute effector functions, including production of antitumor cytokines and killing of target cells, thus acting as living drugs that could potentially have both immediate and long-term effects^6^. Different suitable glioblastoma-specific cell surface antigens have been identified as targets for CAR T cell therapy, such as the epidermal growth factor receptor vIII (EGFRvIII), HER2, IL-13 receptor α chain 2 (IL13Rα2), B7-H3, including disialoganglioside GD2 which have undergone both animal trials and subsequent clinical trials^7–9^.

In this study, we propose disialoganglioside GD2 antigen as a valuable target for CAR T cell therapy to treat glioblastoma. Interestingly, it has been demonstrated that glioblastoma cell lines and primary biopsies commonly express high levels of GD2 antigen^10–14^. On the other hand, GD2 is a relatively minor component of the normal central nervous system, comprising 1–2% of the total amount of gangliosides^15^, therefore representing a potential clinical target for glioblastoma and other brain tumors. We have previously investigated the efficacy of GD2 CAR T cells in a preclinical model of neuroblastoma, highlighting a specific, robust antitumor activity in vitro and in vivo^16^. In the current study, GD2 CAR T approach has been challenged in vitro and in vivo against glioblastoma lines, patient-derived cells and, for the first time, in an autologous setting in vitro.

## Results

### Patient-derived cells and glioblastoma specimens express high levels of GD2

**Fig. 1:**
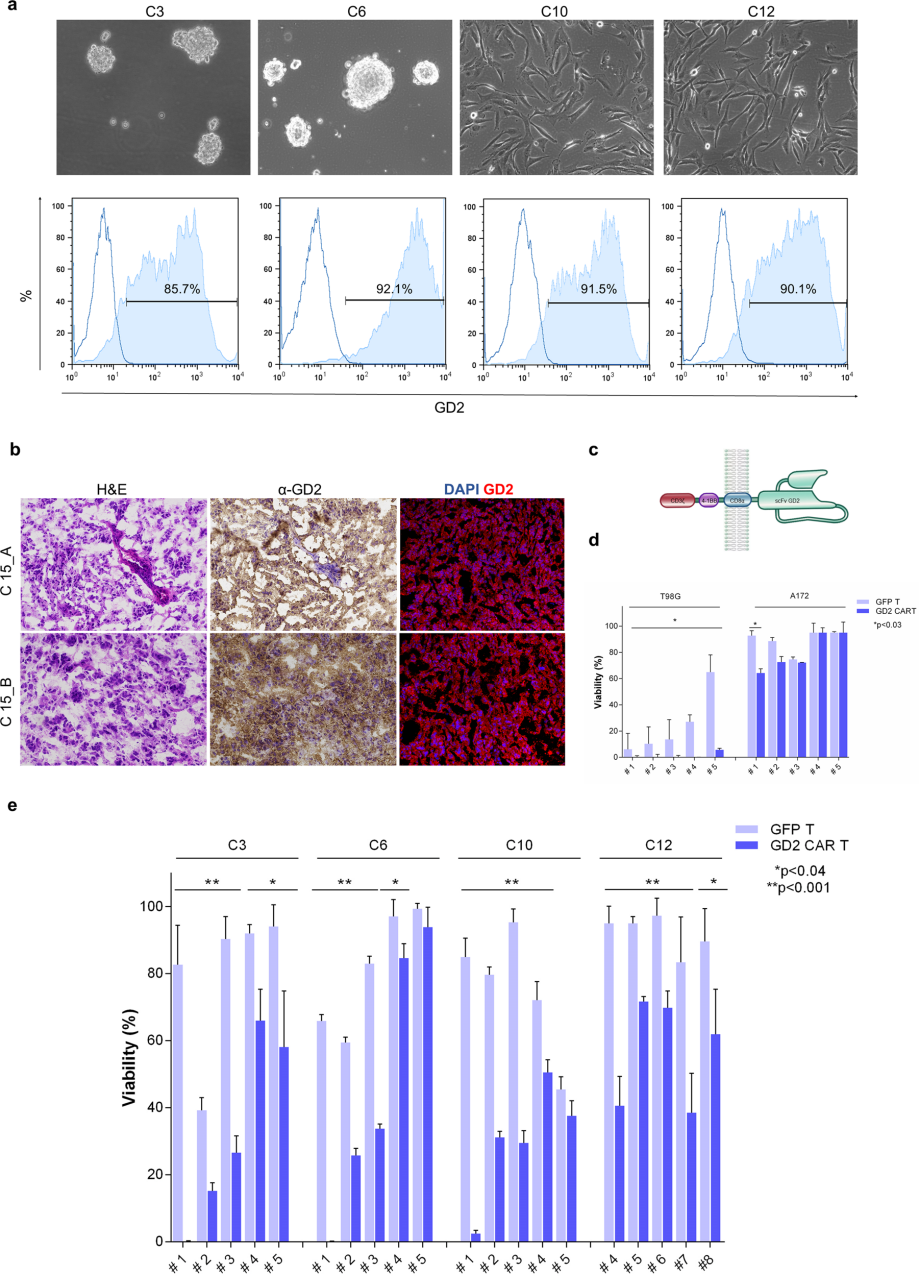
Patient-derived cells and glioblastoma specimens express GD2 and can be targeted by anti-GD2 CAR T cells in vitro. **a** Representative phase contrast microscopy pictures of patient-derived primary glioblastoma cells (C3, C6, C10, and C12) showing different morphological features (upper row). GD2 surface expression (solid histograms) in selected primary glioblastoma samples and isotype control (open histograms) measured by flow cytometry (lower row). **b** GD2 is extensively expressed in patient tumor resections by immunohistochemistry (middle column) and immunofluorescence (right column) stains, on the left column H&E staining. **c** Cartoon representing the second-generation anti-GD2 CAR structure. **d** Glioblastoma cell lines (dsRED T98G GD2^high^, dsRED A172 GD2^low^) and **e** patient-derived primary glioblastoma cells (dsRED C3, dsRED C6, dsRED C10, and dsRED C12) co-cultures at 2:1 E:T ratio with GD2 CAR T and GFP control T cells derived from five PBMC donors (#*n*). After 48 h tumor cell viability is calculated by fluorescence viability assay as reported in “Methods”. Data are shown as mean ± SD from six technical replicates; *p* values are calculated by an unpaired two-tailed *t*-test.

Primary cells were successfully isolated from 12 of 13 tumor resections obtained from glioblastoma grade IV (WHO 2016) specimens with diagnoses confirmed by standard histopathological analyses. After isolation, cells having different in vitro morphological features (Fig. 1a, upper panels) were evaluated for GD2 expression with a positivity greater than 50% in all lines. With 7 out of 12 samples, there was a positivity greater than 80% (Fig. 1a, lower panel). Furthermore, GD2 was evaluated in two commercially available glioblastoma cell lines^14^, T98G (97 ± 1%, GD2^high^) and A172 (2 ± 1%, GD2^low^). Isolated primary glioblastoma cells and cell lines were then successfully transduced by either dsRED retroviral particles or FFLuc lentiviral particles (>80% in all the samples, not shown). In addition, GD2 antigen was confirmed also in glioblastoma resections showing an in vivo extensive expression of the target (Fig. 1b).

### GD2 CAR T cells mediate antitumor activity against GD2-positive glioblastoma cells

After transduction and expansion, the modified T cell population resulted in a mix of CD4^+^/CD8^+^ cells (Supplementary Fig. 1). The cytotoxic activities of GD2 CAR T (structure in Fig. 1c) and GFP T control (transduction efficiency detected by GFP signal, 60 ± 10% and 75 ± 15%, respectively) were assessed over different periods, distinct co-culture conditions, and using different peripheral blood mononuclear cell (PBMC) donors to account for inter-donor variability as reported^17^, as well as targeting selected primary glioblastoma cells (C3, C6, C10, and C12). Firstly, we evaluated the CAR T killing activity against the two cell lines (T98G GD2^high^ and A172 GD2^low^) after 48 h of co-culture at an effector:target (E:T) ratio of 2:1 (Fig. 1d). General allogenic response was observed against T98G GD2^high^, in particular for donor #1, #2, #3, and #4 highlighting an intrinsic immune sensitivity in this cell line. However, on top of the allogenic response, GD2 CAR T cells led to a massive clearance of the tumor within 48 h. On the contrary, A172 GD2^low^ control cell line was minimally affected by both allogenic and anti-GD2 CAR activities, except for donor #1.

To further challenge CAR T potential in a more reliable preclinical model, GD2 CAR T cells derived from different PBMC donors were co-cultured with selected GD2-positive dsRED primary glioblastoma cells (C3, C6, C10, and C12). The viability of all primary glioblastoma cells was significantly reduced in co-culture with GD2 CAR T cells at an E:T ratio of 2:1 in comparison to the parental GFP T cells after 48 h, with antitumor inter-individual variability among PBMC donors. Interestingly, we observed that donor #1 exerted the most dramatic anti-GD2 CAR killing associated with a relatively low allogeneic response. Moreover, we have to report an intra-individual variability, particularly referring to donor #5, who displayed specific activity toward C3 and C12 (Fig. 1e).

**Fig. 2:**
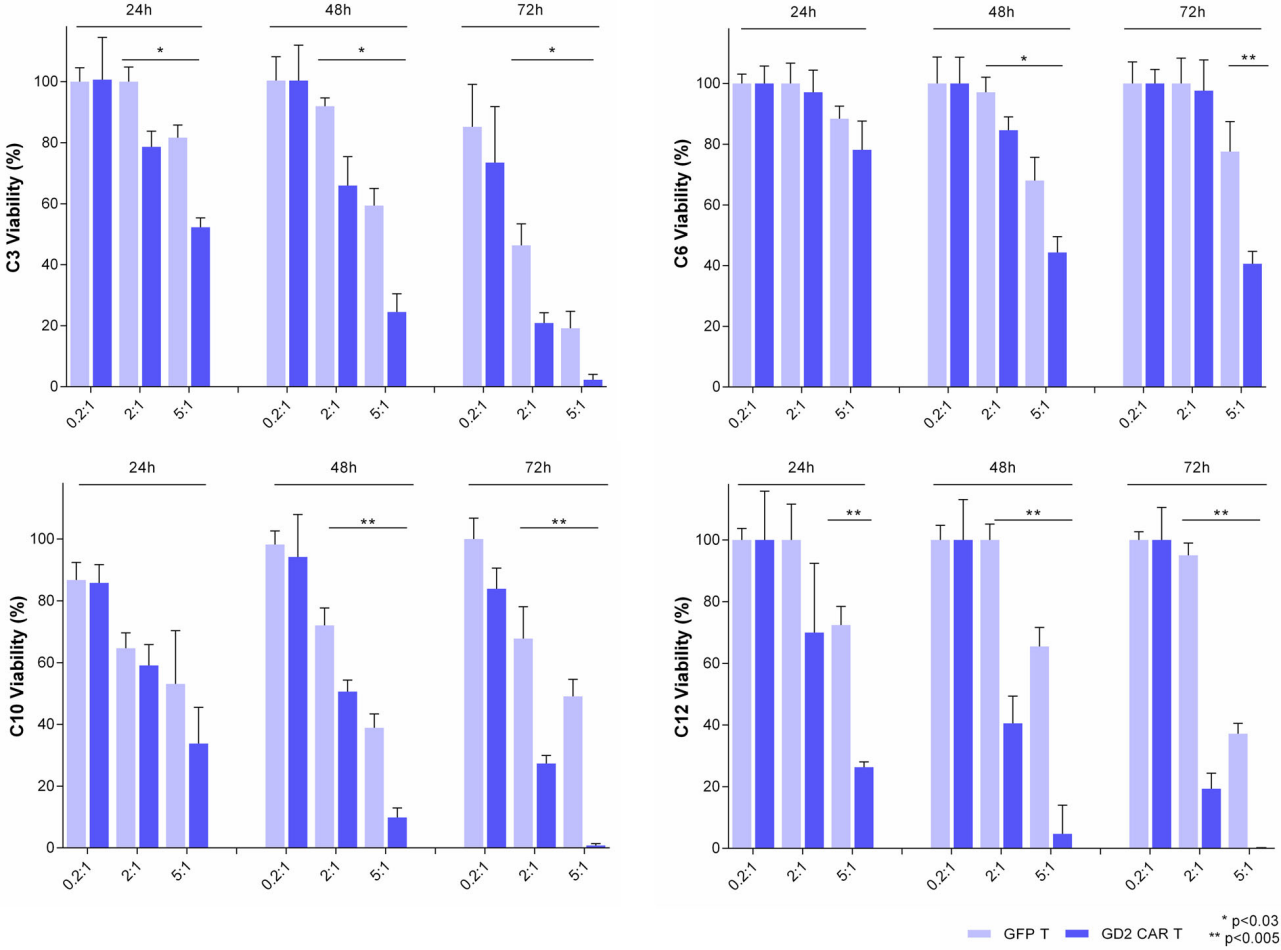
GD2 CAR T cells exert specific killing in a time- and dose-dependent manner against several GD2^high^ patient-derived primary glioblastoma cells. Glioblastoma cells (dsRED C3, dsRED C6, dsRED C10, and dsRED C12) co-cultures with GD2 CAR T and GFP control T cells at three different E:T ratios. After 24, 48, and 72 h tumor viability is calculated by fluorescence viability assay as reported in “Methods”. Representative bar graphs from PBMC donor #4 are reported. Data are shown as mean ± SD from six technical replicates; *p* values are calculated by unpaired two-tailed *t*-test.

**Fig. 3:**
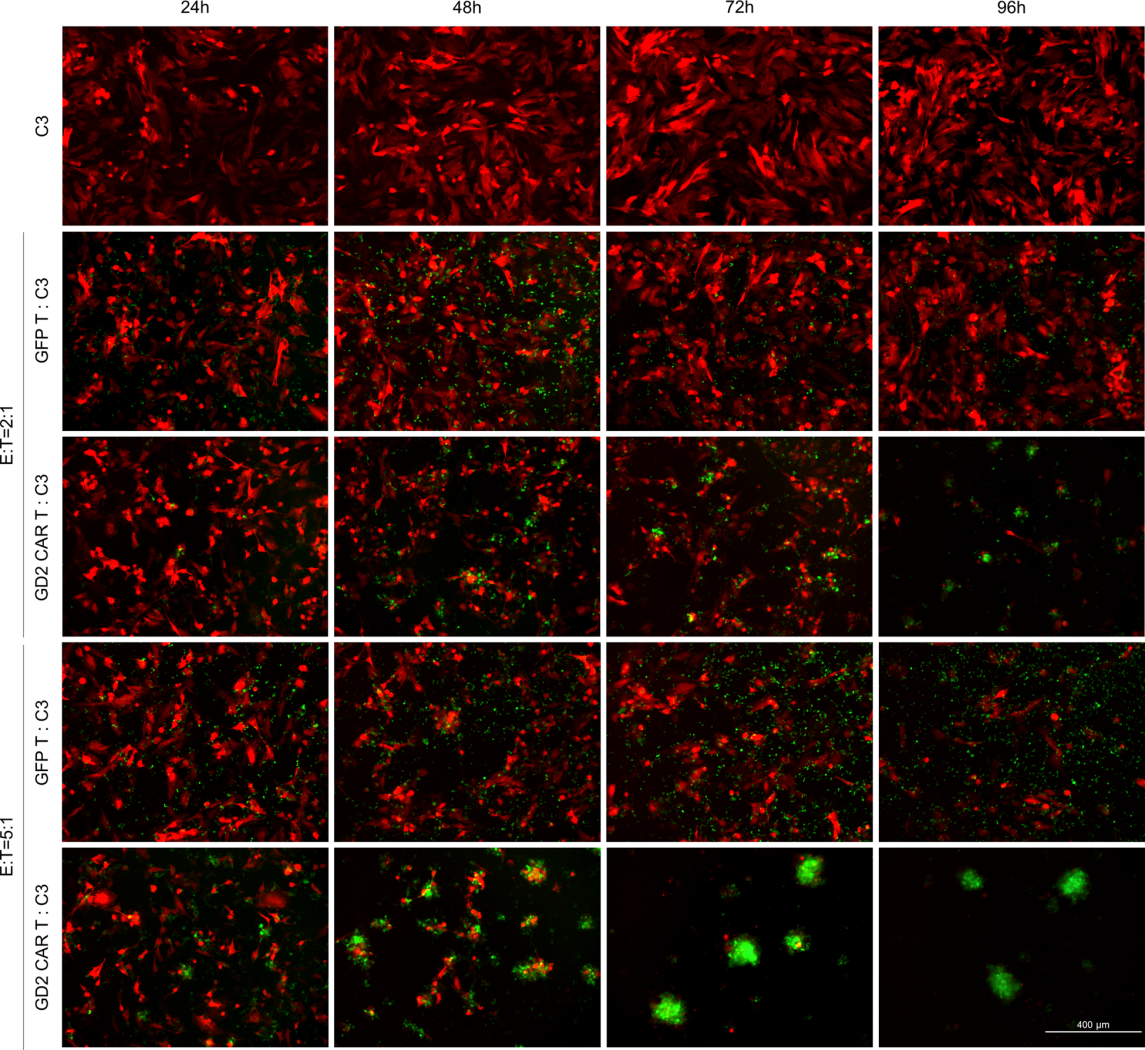
GD2 CAR T cells generate clusters to eradicate patient-derived primary glioblastoma cells. Representative fluorescence micrographs highlight a robust cluster activation and killing activity of GD2 CAR T population against primary glioblastoma cells. This feature was never evident in GFP T control cells. DsRED C3 glioblastoma (red) cells in co-culture with GD2 CAR T (green) and GFP T control cells (green) at 2:1 and 5:1 E:T ratios at 24, 48, 72, and 96 h. The scale bar: 400 µm.

Once target specificity at 48 h was defined, to further address the GD2 CAR T cytotoxic effect over different periods (24/48/72 h) and co-culture E:T ratios (0.2:1, 2:1, 5:1), donor #4 was selected as mid-performer (89 ± 11% vs 60 ± 19% tumor viability in co-culture with GFP T and GD2 CAR T, respectively; *t*-test *p* = 0.043) among PBMC donors in terms of GD2 CAR antitumor activity. Overall, except for the lowest E:T ratio of 0.2:1, cytotoxic activity of CAR T cells was significantly higher compared to the control GFP T cells for all primary glioblastoma cells in a time- and dose-dependent manner (Fig. 2). For this purpose, we reported a representative fluorescence imaging of co-cultures over time at ratios of 2:1 and 5:1 E:T (Fig. 3). Specifically, GD2 CAR T at a 5:1 ratio surrounded dsRED primary glioblastoma line C3 forming large and reactive T cell clusters (green) that progressively eliminated target cells (Fig. 3, bottom row). Similarly, antitumor activity was observed at a 2:1 ratio, although the effect was delayed in time (Fig. 3, third row). On the contrary, no reactive clusters were observed in GFP T control condition showing tumor cells not affected by cytotoxic activity over time (Fig. 3, second and fourth row). Collectively, these data indicate the capacity of anti-GD2 CAR to elicit a specific, dose-dependent antitumor response against primary glioblastoma cells.

**Fig. 4:**
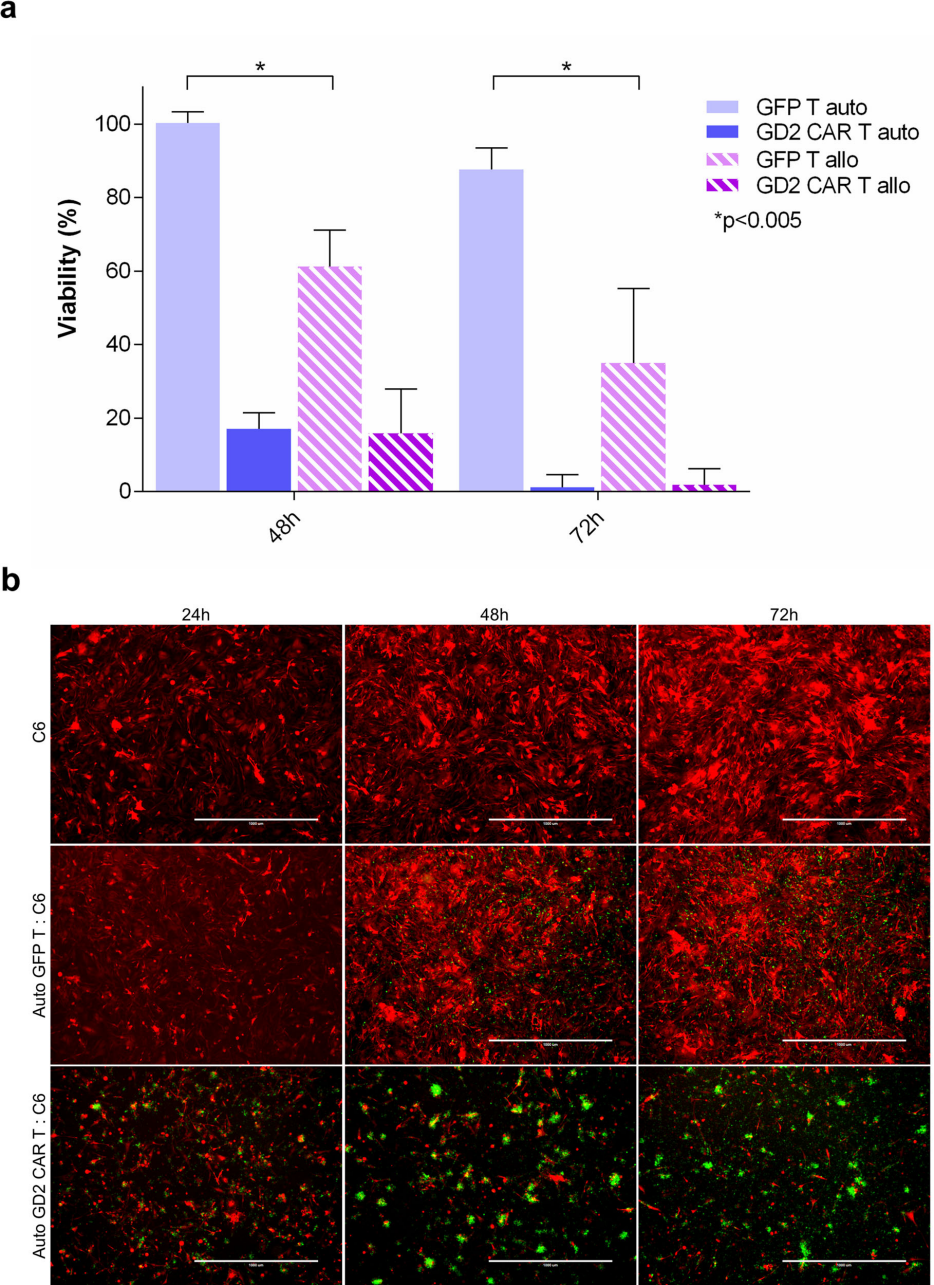
Autologous GD2 CAR T cells kill glioblastoma in vitro. **a** Glioblastoma cells (dsRED C6) co-cultures with either autologous or allogeneic GD2 CAR T and GFP control T cells at 5:1 E:T ratios. After 48 and 72 h tumor viability is calculated by fluorescence assay as reported in “Methods”. Data are shown as mean ± SD from three technical replicates; *p* values are calculated by unpaired two-tailed *t*-test. **b** Representative fluorescence micrographs show clusters of activation and killing activity of GD2 CAR T cells. GFP T cell population show an absence of reactivity of autologous cytotoxic lymphocytes. DsRED C6 glioblastoma (red) cells in co-culture with GD2 CAR T (green) and GFP T control cells (green) at 5:1 E:T ratio at 24, 48, and 72 h. The scale bar: 1000 µm.

Originally, CAR T approaches have been mainly conceived within an autologous setting. For this reason and in order to address the allogeneic background related to healthy donor T cells, GD2 CAR T approach was additionally challenged with autologous cells (Fig. 4). DsRED patient-derived glioblastoma cells (C6) were targeted in co-culture assays either with patient-derived autologous or with allogeneic GD2 CAR T cells at an E:T ratio of 5:1 for 48 and 72 h (Fig. 4a).

As previously shown (Fig. 2), allogeneic GD2 CAR T cells exerted an important tumor killing associated with allogenic background in the GFP T counterpart. Interestingly, the introduction of GD2 CAR in autologous T cells was able to reproduce the same dramatic anticancer effect with cluster of activated cytotoxic lymphocytes (Fig. 4a, b). On the contrary, autologous GFP T cells did not significantly affect tumor viability in comparison to the allogeneic GFP T cells confirming that the CAR introduction drives a specific and robust anti-glioblastoma effect in the autologous setting.

Since CAR T cells are reported to undergo in the exhaustion phase^18^, we decided to perform a second round of tumor killing with previously tumor-primed T cells in comparison to naïve parental cells (Supplementary Fig. 2a, b). Naïve CAR T cells exerted their antitumor activity as expected. Interestingly, primed CAR T lymphocytes were not exhausted and conversely generated a potent cytotoxic effect starting even earlier than naïve T cells (Supplementary Fig. 2b). Collectively these data showed the in vitro potential of both allogeneic and autologous GD2 CAR T cells against glioblastoma, even within a rechallenge assay.

### GD2 CAR T activity is selectively associated with cytokine release not impacted by TGF β1

**Fig. 5:**
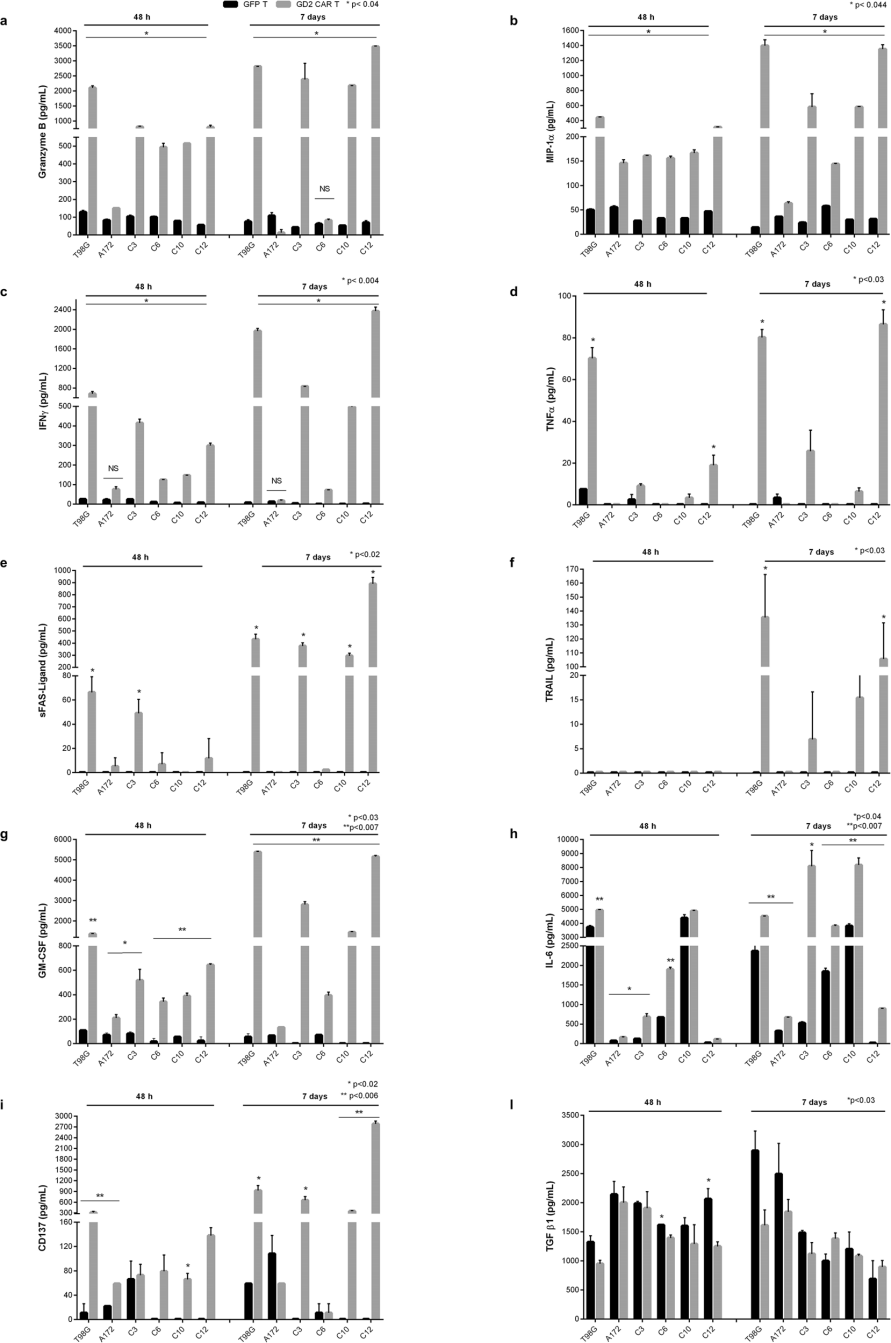
Cytokine profile during GD2 CAR T cells in co-culture with glioblastoma cells. Co-culture supernatants of glioblastoma cell lines (dsRED T98G GD2^high^, dsRED A172 GD2^low^) and primary glioblastoma cells (dsRED C3, dsRED C6, dsRED C10 and dsRED C12) with either GD2 CAR T or GFP control T cells (donor #4) at 2:1 E:T ratio are assessed for cytokine release at 48 h and 7days. Cytokine profile is measured by multiplex Luminex system, and the concentration level is reported as pg/mL. **a** Granzyme B. **b** MIP-1α. **c** IFNγ. **d** TNFα. **e** sFAS-Ligand. **f** TRAIL. **g** GM-CSF. **h** IL-6. **i** CD137. **l** TGF β1. Data are shown as mean ± SD from two technical replicates; *p* values are calculated by unpaired two-tailed *t*-test. NS not significant.

In the attempt to dissect biomolecules involved in CAR T and glioblastoma interplay, cytokine production was evaluated in supernatants at both 48 h and 7 days. Specimens were obtained from co-cultures of either GD2 CAR T or GFP T control cells (donor #4) with target cells. Specifically, granzyme B and macrophage inflammatory protein-1α (MIP-1α) were consistently released (48 h and 7 days) at higher levels in GD2 CAR co-cultures versus controls except for A172 GD2^low^ at 7 days (Fig. 5a, b). Similarly, IFNγ, TNFα, and sFAS-ligand increased versus control at 48 h and then peaked after 7 days (Fig. 5c, d, e). Again, anti-A172 GD2^low^ cytotoxicity was associated with low or absent levels of IFNγ, TNFα, and sFAS-ligand with no differences between GD2 CAR T versus GFP T control. Interestingly, TRAIL levels increased only at 7 days in all the samples treated with GD2 CAR T cells, except for A172 GD2^low^ and C6 primary line (Fig. 5f). We also considered pro-inflammatory cytokines, such as GM-CSF and IL-6, as potential key players in CAR T cell mode of action (Fig. 5g, h). GM-CSF was consistently and significantly higher in GD2 CAR T co-cultures versus control. A similar trend was observed for IL-6, whose levels increased after 7 days in all primary glioblastoma samples. We additionally focused on CD137, a costimulatory molecule that was significantly higher over time in GD2 CAR co-cultures versus control, except for A172 GD2^low^ (both at 48 h and 7 days), for C3 line at 48 h, and for C6 primary cells at 7 days (Fig. 5i). We then considered a key immunosuppressant most abundantly expressed by tumors and tumor-associated cells, such as TGF β1, which plays a central role in immunomodulation and tumor escape^19–21^. We observed that TGF β1 was consistently expressed in all considered conditions either at 48 h or at 7 days with no difference between GD2 CAR T and GFP T control cells (Fig. 5l). These data indicate a general CAR-restricted release of effector and pro-inflammatory cytokines that take place over time. Most importantly, cytokine release does not arise in A172 GD2^low^ co-cultures with GD2 CAR T cells, suggesting that GD2 expression in tumor cells represents a key condition to trigger an organized immune-response to generate a therapeutic benefit.

### GD2 CAR T cytotoxic activity is preserved in two distinct three-dimensional glioblastoma models

**Fig. 6:**
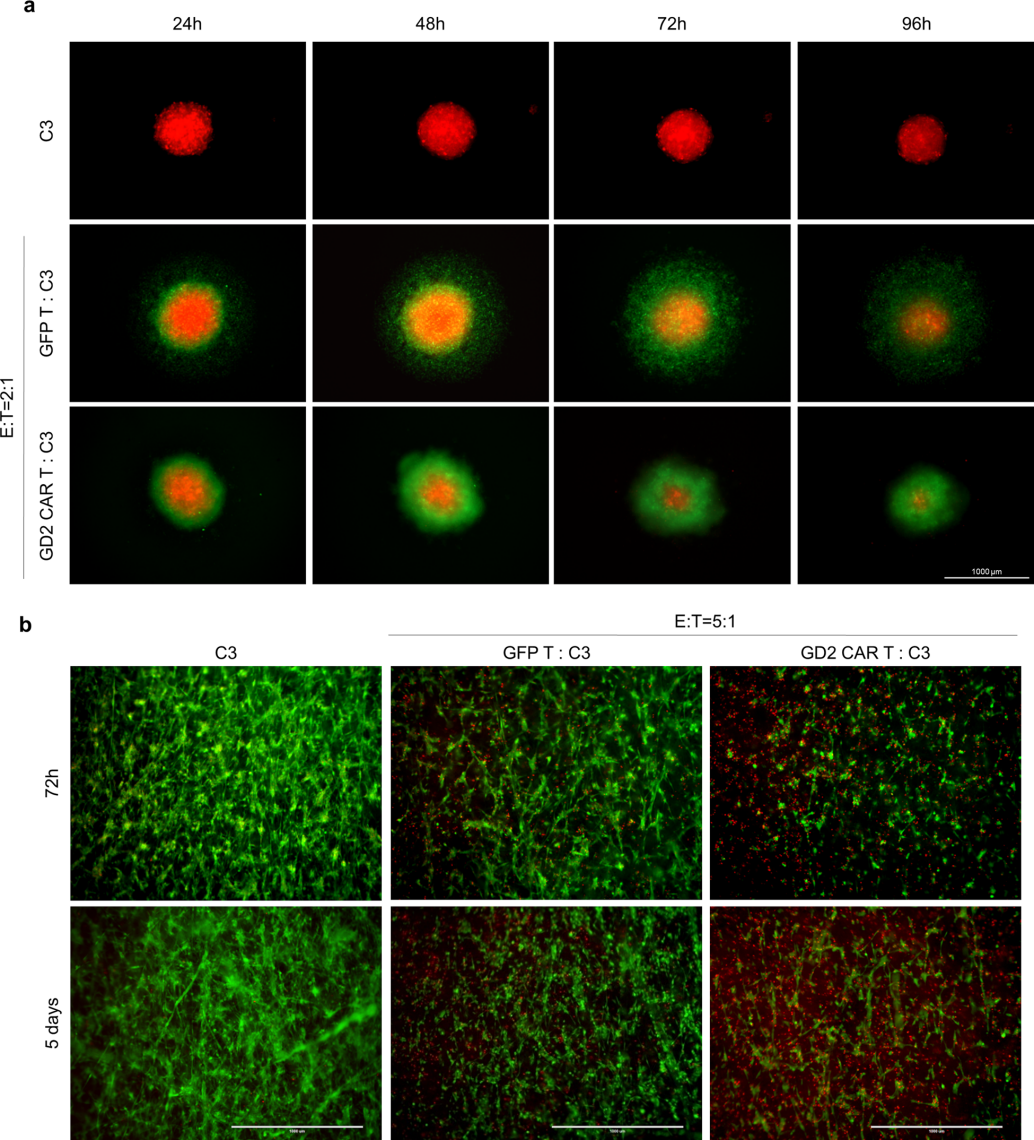
Three-dimensional in vitro models confirm GD2 CAR T specific action against glioblastoma. **a** Representative fluorescence microscopy images of glioblastoma spheroids alone (upper row) or in co-culture with either GD2 CAR T (bottom row) or GFP control T cells (middle row) at 2:1 E:T ratio. CAR T cells antitumor activity is monitored at 24, 48, 72, and 96 h. Tumor cells in red, GD2 CAR T cells and GFP control T cells in green. The scale bar is 1000 µm. **b** Representative fluorescence microscopy images of primary C3 glioblastoma cells forming a tissue-like structure in VITVO^®^ 3D bioreactor at 72 h and 5 days (left row) and then in co-culture with GD2 CAR T (right row) and GFP control T cells (middle row) at 5:1 E:T ratio. A fluorescence-based live/dead assay is used to monitor the cytotoxic effect of CAR T cells at 72 h and 5 days (living cells in green, dead cells in red). The scale bar: 1000 µm.

Tumors develop within complex cell-to-cell interactions. In this view, three-dimensional (3D) cultures may help to understand the tumor development accounting for the spatial distribution and challenges faced by immune cells in infiltrating a solid bulk. Thus, two different 3D tumor models were used to challenge the anti-GD2 CAR strategy. In the first one, tumor spheroids of dsRED C3 glioblastoma sample were co-cultured with CAR T cells at an E:T ratio of 2:1. In this context, the antitumor activity was characterized by highly reactive T cell clusters localized to the tumor spheroid whose dimension was progressively reduced over time in co-culture. Conversely, GFP T cell population displayed a more dispersed localization around the tumor spheroid appearing marginally affected by the effector-killing activity (Fig. 6a). To further address GD2 CAR T antitumor performance, we used a biocompatible 3D cell culture device that allows a rapid ex vivo rebuilding of the tumor’s tissue-like structure^22^. Thus, C3 tumor cells were loaded and observed rapidly colonizing the 3D matrix and after 24 h either GD2 CAR T or control GFP T cells were added to the 3D culture at an E:T ratio of 5:1. Fluorescence microscopy monitoring, using a live (green cells) and dead (red cells) kit, at 72 h and at 5 days showed an evident cytotoxic effect, mediated by GD2 CAR T cells compared to either the control counterpart or the tumor alone (Fig. 6b). These data indicate that in both 3D glioblastoma models, GD2 CAR T cell potential is maintained, suggesting a specific dynamic of interaction in vitro as a prerequisite to test their in vivo performance.

### Intracerebral GD2 CAR T cells improve survival in orthotopic glioblastoma model

**Fig. 7:**
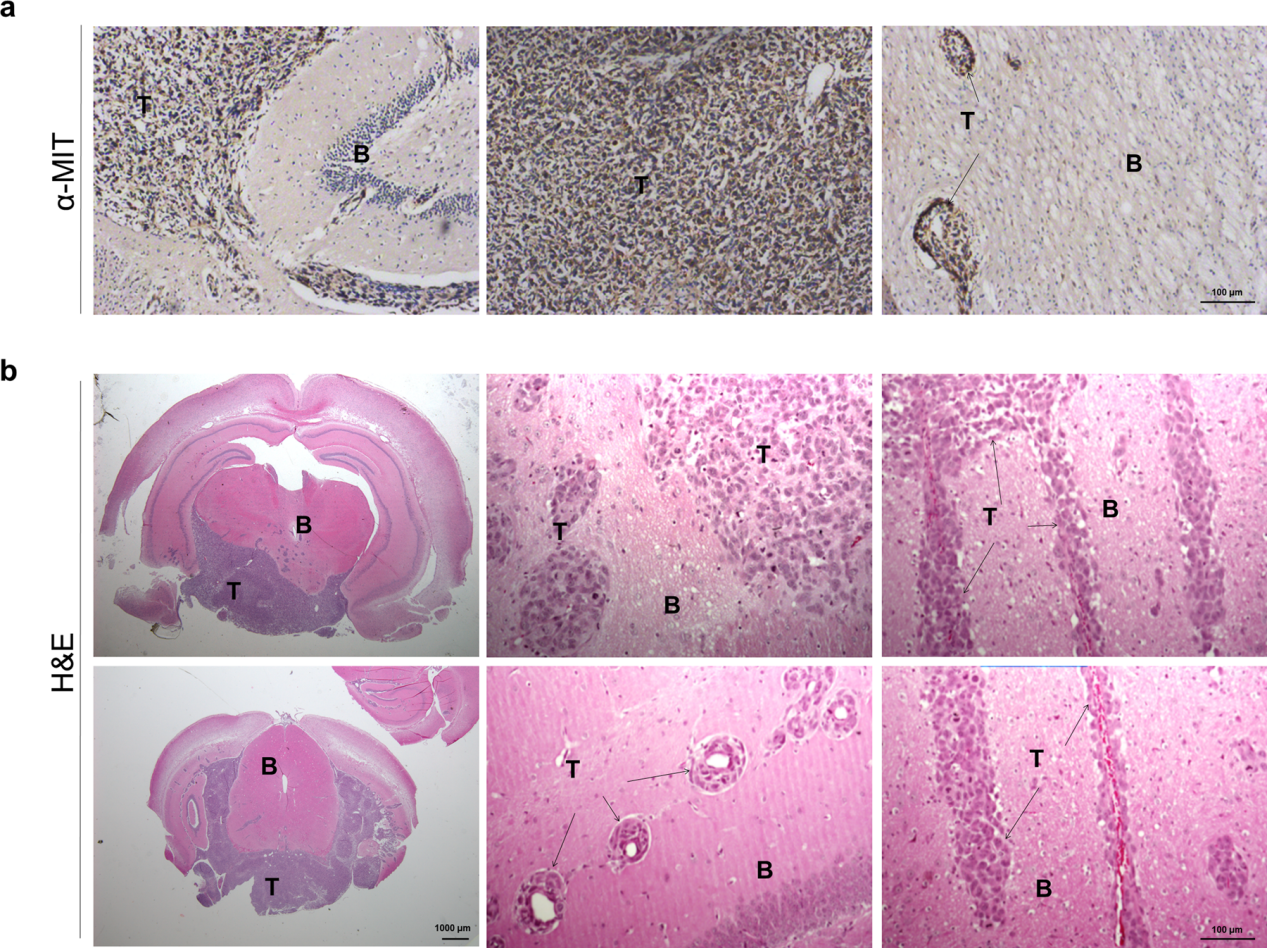
Primary patient-derived cells engraft in an orthotopic NOD/SCID model recapitulating the infiltrative nature of glioblastoma. **a** Representative images of histological specimens immune-labeled with anti-human mitochondrial (MIT) monoclonal antibody (brown: DAB) confirmed the engraftment of intracranial injected patient-derived glioblastoma C12.FFLuc cells (T) in mouse brain (B). The scale bar: 100 µm. **b** Hematoxylin and eosin staining show the high infiltrative capacity of C12.FFLuc tumor cells (T) in mouse brain (B). First column, the scale bar is 1000 µm. Second and third columns, the scale bar: 100 µm. All sections were derived from formalin-fixed paraffin-embedded brains of euthanized mice at the initial signs of distress and weight loss of up to 30%.

To further validate GD2 CAR T strategy, we generated an orthotopic model using primary glioblastoma cells (C12 GD2-positive.FFLuc) transduced by a luciferase-expressing vector for in vivo monitoring. This model resulted in a rapid growth rate that was consistently lethal within 28–30 days after injection of 1 × 10^5^ cells. Animal brains were then analyzed postmortem with evidence of anti-human mitochondrial positive cells (MIT^+^), confirming the human nature of cells infiltrating the murine parenchyma (Fig. 7a). The further histologic evaluation demonstrated glioblastoma cells’ ability to grow in an already highly vascularized organ such as the brain and to exhibit diffuse invasion of the surrounding tissue. Interestingly, we observed that tumor cells were able to localize along blood vessels (Fig. 7b). This feature has been already described as “vessel co-option”^23^, where glioblastoma cells move toward preexisting blood vessels, taking advantage of oxygen and nutrient supply as well as employing vasculature as a scaffold from which to invade normal tissue.

The generation of this model recapitulated the infiltrative nature of glioblastoma; thus, we established two strategies to evaluate in vivo efficacy of GD2 CAR T cells. In the first strategy, mice orthotopically injected with C12.FFLuc GD2-positive cells were treated by intravenous (IV) doses of 1.5 × 10^6^ at day 8 and 1.9 × 10^6^ at day 20 post-intracranial tumor injection with either GD2 CAR T cells or control GFP T cells (Supplementary Fig. 3a). After IV infusion there was no substantial survival-rate increase in mice treated with GD2 CAR T cells with respect to the group treated with either GFP T cells or PBS control; thus, animals were invariably sacrificed at day 28 due to massive tumor progression (Supplementary Fig. 3b). We further challenged the IV administration with the higher dose (5 × 10^6^) of CAR T cells at days 7 and 14 post-intracranial tumor injection (Supplementary Fig. 3c). Again, we have to report the lack of efficacy by IV administration in our model (Supplementary Fig. 3d). This may be due to either the incapability of modified T cells to reach intracerebral glioblastoma or to the possible downregulation of GD2 signal in vivo, as then addressed by immunofluorescence and immunohistochemistry studies (Supplementary Fig. 3e). After IV infusion, T cells seem incapable to localize and infiltrate the tumor mass (Supplementary Fig. 3e). Regarding GD2 expression, after tumor explant, we showed extensive and persisting antigen presence in IV treatment (Supplementary Fig. 3f).

**Fig. 8:**
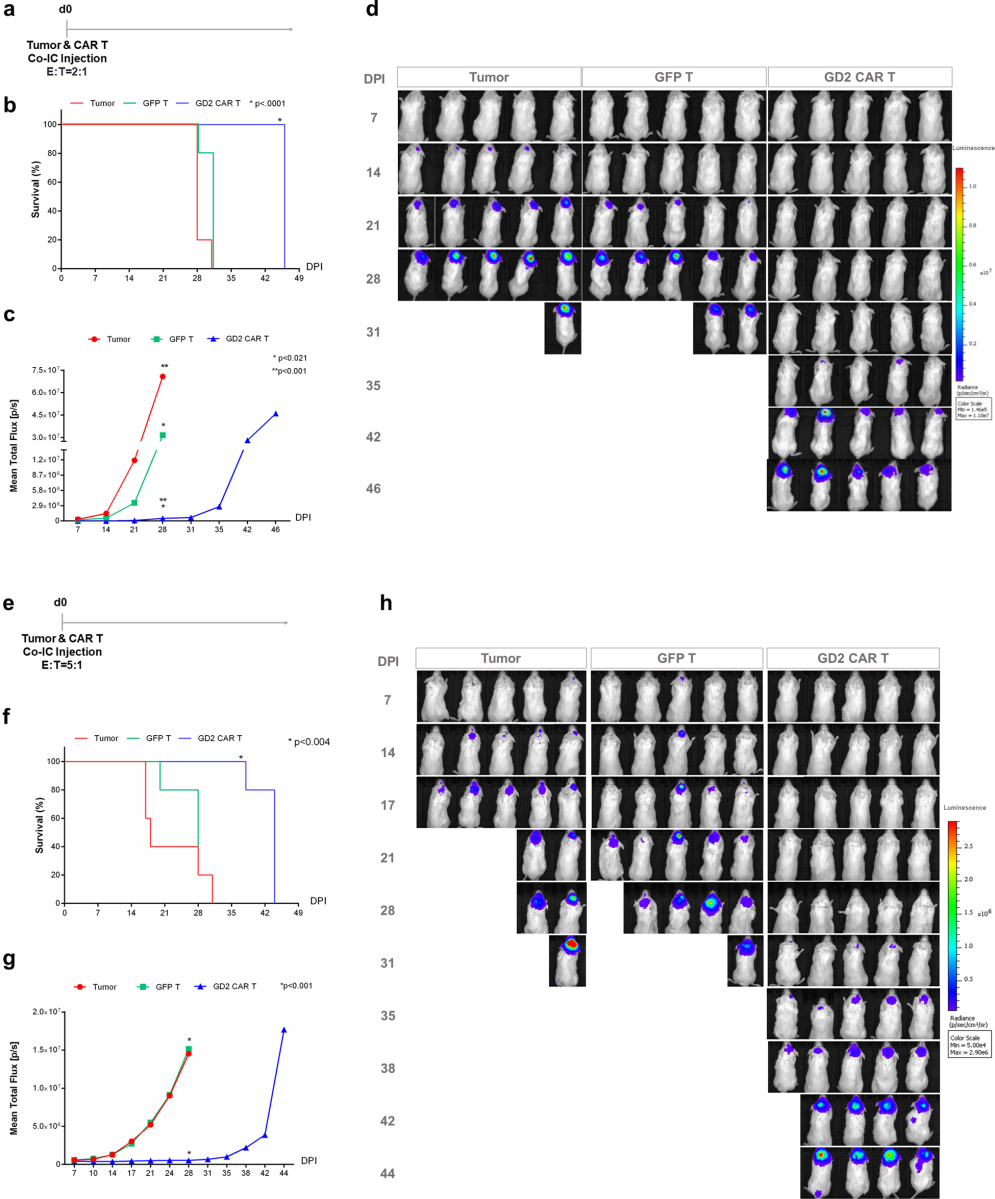
Intracerebral GD2 CAR T cell treatment improves survival in the orthotopic glioblastoma model. **a** Schematic outline of CAR T treatment by intracerebral (IC) co-injection. NOD/SCID mice (*n* = 5) are co-injected intracranially with 1 × 10^5^ C12.FFLuc cells and 2 × 10^5^ of either GD2 CAR T cells or GFP control T cells at day 0. **b** Kaplan–Meier survival curve and log-rank tests are used to measure differences among groups. **c** Mean total flux values (photons/s) of either GD2 CAR T or GFP T control-treated mice and control animals monitored by IVIS system. Data are shown as a mean ± SD; *p* values are calculated by unpaired two-tailed *t*-test. **d** Tumor progression is monitored weekly by a luminescence signal with IVIS imaging measuring. DPI day post-injection. **e** Schematic outline of CAR T treatment by intracerebral (IC) co-injection. NOD/SCID mice (*n* = 5) are IC co-injected with 1 × 10^5^ C12.FFLuc cells and 5 × 10^5^ of either GD2 CAR T cells or GFP control T cells at day 0. **f** Kaplan–Meier survival curve and log-rank tests are used to measure differences among groups. **g** Mean total flux values (photons/s) of either GD2 CAR T or GFP T control-treated mice and control animals monitored by IVIS system. Data are shown as a mean ± SD; *p* values are calculated by unpaired two-tailed *t*-test. **h** Tumor progression is monitored weekly by a luminescence signal with IVIS imaging measuring. DPI day post-injection.

The intracerebral (IC) co-injection of a single dose of 2 × 10^5^ GD2 CAR T cells along with 1 × 10^5^ C12.FFLuc GD2-positive cells (Fig. 8a) led, on the contrary, to a substantial and significantly improved survival rate in the GD2 CAR-treated group with respect to controls (Fig. 8b, c, d). The median survival period was 28 days for mice without any treatment, 31 days for the GFP T cell-treated group, and 46 days for GD2 CAR–treated animals. One single dose of GD2 CAR T treatment at an E:T ratio of 2:1 was enough to control glioblastoma growth with an average delay in tumor progression of 2 weeks. Specifically, we observed 1.6- and 1.5-fold change in survival time of GD2 CAR T-treated animals versus untreated and GFP T-treated mice, respectively. Ultimately, mice were sacrificed at day 46 for ethical reasons due to tumor progression. As for the IV model, the IC co-injection treatment was further challenged at a higher dose using 5 × 10^5^ GD2 CAR T cells along with 1 × 10^5^ C12.FFLuc GD2-positive cells (Fig. 8e). A single dose of GD2 CAR T at the E:T ratio of 5:1 determined a greater control on glioblastoma growth with an average delay of up to 4 weeks. Specifically, we observed a significant 2.4- and 1.6-fold change in survival time of GD2 CAR T-treated mice versus untreated and GFP T-treated mice, respectively (Fig. 8f, g, h). Immunofluorescence and immunohistochemistry studies demonstrated that at sacrifices glioblastoma cells infiltrate brain tissue (Supplementary Fig. 4a, upper row), showing persistent GD2 expression (Supplementary Fig. 4a, middle and lower rows). Interestingly, we could observe the persistence of several GFP^+^ T cells in the IC treatment mice (Supplementary Fig. 4b). Importantly, the four (both IV and IC) treatments were not associated with visible systemic and neurologic side effects.

## Discussion

The current standard of care for glioblastoma still provides an unacceptable prognosis. Despite a variety of novel treatment modalities investigated for glioblastoma—e.g. immune checkpoint inhibitors, oncolytic viruses, and vaccines—there has been limited success in treating both primary and recurrent glioblastoma^5,24^. Gene therapy and immunotherapy have shown promise in potentially revolutionizing treatment for a variety of cancers^24,25^. However, so far, this potential has not been realized for the treatment of glioblastoma, which still stands as a problematic and deadly form of cancer.

Immunotherapies involving CAR T have demonstrated efficacy in hematologic malignancies with five US FDA-approved CAR T-based therapies (fda.gov). The success achieved with hematology has made possible a host of efforts to develop CAR T cell therapies for solid tumors as well, including glioblastoma. To date, five valuable glioblastoma-specific cell surface antigens have been identified as targets for CAR T cells, such as EGFRvIII, HER2/neu, IL-13Rα2, B7-H3 and EphA2^7^. Some of them underwent animal preclinical testing^26,27^ and then entered into early trials such as EGFRvIII CAR T (NCT01454596, NCT02664363, NCT02209376), IL-13Rα2 CAR T (NCT02208362), HER2 CAR T (NCT02442297, NCT01109095), and EphA2 CAR T (NCT02575261)^28^. Although completed trials for glioblastoma with anti-EGFRvIII CAR T (NCT02209376)^29^, (NCT01454596)^30,31^, anti-HER2 CAR T (NCT01109095)^32^, and ongoing trials with anti-IL-13Rα2 CAR T (NCT02208362)^33,34^ reported promising results, there are still several safety concerns to be addressed and areas that need further improvement^35^. Brown et al.^34^ published a case report of a recurrent glioblastoma patient treated with IL13Rα2 CAR T cells demonstrating significant clinical and radiographic response, although recurrence occurred 7.5 months post-treatment. O’Rourke et al.^29^ reported an acceptable safety profile and CAR T infiltration of the tumor in 10 patients with recurrent glioblastoma treated with EGFRvIII CAR T cells, although with no survival benefit. Ahmed et al.^32^ reported an acceptable safety profile for 16 patients treated with HER2 CAR T cells, achieving a partial response and stabilizing cancer for 8 weeks to 29 months.

Despite these early exciting clinical results, glioblastoma still has several unique features in terms of tumor heterogeneity with the associated immunosuppressive environment, antigen escape, and physical barriers that may limit CAR T-based strategies. In addition, it is not yet clear which is the optimal delivery route for CAR T cell administration; to date, IV, IC, and intraventricularly (IVT) administrations have been attempted^19^. Among them, IVT is emerging as the most effective approach in at least one case where a significant regression of glioblastoma was demonstrated^34^. However, the significance of these findings is limited to a single patient, which would suggest the need for further investigation.

The current study proposes GD2 as a target for CAR T cell therapy in in vitro and in vivo preclinical models of human glioblastoma targeting primary cancer cells and the first to attempt an autologous setting versus GD2 as target. GD2 has been identified by a few research groups as target for CAR T strategy for cerebral tumors^8,36,37^. In particular, Zimmermann et al.^8^ supported an in vitro approach to enhance CAR T action with GD2-positive patient-derived primary glioblastoma cells. Mount et al.^36^ demonstrated potent antitumor characteristics of GD2 CAR T cells in vitro and in vivo on patient-derived diffuse intrinsic pontine glioma (DIPG) and other human diffuse midline gliomas with mutated histone H3 K27M. On the other hand, Murty et al.^37^ reported for the first time longitudinal fluorescence-based intravital microscopy imaging of murine GD2 CAR T cells within syngeneic orthotopic models of glioblastoma, demonstrating the synergistic effect of CAR T cells and radiation therapy. On clinical data, two recruiting phase I clinical trials are currently ongoing (NCT04099797, NCT04196413) for patients with high-grade glioma (HGG) or DIPG; still, however, there are no clinical trials on GD2 as glioblastoma target. So far, GD2 CAR T approach for glioblastoma has been partly considered despite the clinical data on their safety in targeting neuroblastoma^38^. This is also in line with our previous preclinical data on a neuroblastoma model which show a robust antitumor activity without evidence of off-tumor toxicity^16^.

Starting from this basis, our effort has been to further challenge the anti-GD2 CAR approach with a variety of patient-derived GD2-positive glioblastoma cells within several two-dimensional (2D) and 3D in vitro assays and in two distinct in vivo studies. Moreover, we have introduced an autologous setting to treat GD2-positive primary glioblastoma cells, as previously reported for anti-HER2 CAR T cells^26^.

We report that patient-derived glioblastoma cells exhibit a consistent and high expression of GD2 representing a valuable target for glioblastoma. Samples are characterized by a variety of morphologic features reflecting, to some extent, the heterogeneity of glioblastoma multiforme^39^. Despite an inter-donor T cell variability in killing performance, in vitro data showed that GD2 CAR T cells had the ability to mediate significant and specific antitumor activity with respect to the control GFP T cells and in a time- and dose-dependent manner against both primary glioblastoma samples and cell lines assessed. Considering the experimental allogeneic setting, we have to report an allogenic response background in standard 2D cytotoxicity assays for both GFP T controls and GD2 CAR T cells, which could be related to the phenotype of our effector cells comprising a CIK population and to the mismatch of HLA expression^16^. CAR T technology has been mainly conceived as an autologous approach when translated into the clinic. Thus, we challenged that setting in vitro demonstrating the ability of autologous GD2 CAR T cells in mediating a very powerful and specific antitumor response since the very first time point.

Similarly, 3D glioblastoma models confirmed a robust activation of CAR T cells against the tumor mass. Interestingly, we observed the formation of highly reactive clusters capable of infiltrating and reducing the dimension of glioblastoma spheroids leading to tumor clearance over time.

It is well known that cytotoxic T cells can redirect their antitumor activity by two main pathways: granule exocytosis (i.e. granzymes) and the release of death ligands^20,40^. For this reason, we wanted to investigate a variety of cytokines that might be released during CAR T cell activation. Our data highlight a rapid activation of granzyme B in GD2 CAR T co-cultures, where the killing effect is further enhanced due to the subsequent release of proinflammatory cytokines such as MIP-1α and IFNγ, prostimulatory factors such as GM-CSF and death ligands such as TNFα, sFas-ligand, and TRAIL. Regarding CD137 costimulatory molecules, also included in our second-generation CAR, the soluble form was detected in abundance over time in GD2 CAR co-cultures versus control, suggesting a robust CAR effector activation. On the other hand, an important soluble CD137 release may act as a physiological negative feedback mechanism to prevent an overshooting of lymphocyte activation as a result of CD137 co-stimulation^41^. Gliomas have been described as expressing a variety of cytokines that have an important role in tumor proliferation and angiogenesis but can also allow tumors to escape detection from immune surveillance^42^. In this view, we focused our attention on IL-6 and TGF β1 among the investigated cytokines mentioned previously. Interestingly, they were the only ones released on a large scale even by glioblastoma cells not in co-culture with effector T cells (data not shown). Regarding IL-6 pleiotropic nature^42^, it is reported to be secreted in high-grade gliomas, supporting cell invasion and therapeutic resistance^42^ while directly or indirectly counteracting tumor growth when released as proinflammatory cytokine by CAR T cells^43^. Therefore, we can posit that the IL-6 increase observed in our co-culture supernatants of glioblastoma with GD2 CAR T cells might be due to activated CAR T cell release. This peculiar aspect, outlining dual IL-6 functionality in glioblastoma, deserves further investigation. TGF β1 was significantly secreted during both GD2 CAR T cell and control GFP T cell co-cultures. It is often implicated in the inhibition of immune response and correlated with poor prognosis^21^. Despite TGF β1 levels being comparable, only in GD2 CAR T cell co-cultures we could observe a robust cytotoxic effect, suggesting that the proposed GD2 CAR approach is not affected by this immunosuppressive environment, at least in vitro.

Based on the in vitro evidence, we challenged GD2 CAR T cells in patient-derived GD2-positive glioblastoma orthotopic models in order to provide a preclinical assessment of treatment safety and efficacy. At first, we generated a model with consistent and reproducible tumors across animals. Our model exhibited a rapid growth rate and is consistently lethal within 30 days, thus recapitulating the infiltrative and malignant nature of glioblastoma multiforme in histologic studies.

Concerning the delivery route, it could significantly impact the outcome for solid tumors in cell therapies, especially for brain tumors such as glioblastoma^19^. Thus, we compared the route of administration of CAR T cells considering both the IV systemic and the IC in loco delivery. Systemic IV delivery of CAR T cells did not lead to any improvement in the survival rate of CAR T-treated mice with respect to the control groups for both total doses tested of 3.4 × 10^6^ and 10^7^ CAR T cells. Limited to our model and the preclinical observations, the IV delivery has been associated with low T cell infiltration, presumably due to a poor homing and to an immunosuppressive microenvironment in line with reports in clinical and preclinical studies^44–46^.

On the other hand, IC in loco treatment via co-injecting glioblastoma cells, mimicking a minimal residual disease after resection, along with T cells was established by administrating two different doses, leading to a significantly improved survival rate in mice treated with GD2 CAR T cells with respect to either GFP T cell-treated animals or those without any treatments. Indeed, the tumor burden in the treated mice started emerging with a 2-week delay for the lower dose of treatment and with a 3–4 weeks for the higher dose, associated with intra-tumor persistence of modified T cells. Importantly, both systemic and in loco treatments were not associated with cytokine release syndrome or any neurologic toxicities as contrarily reported by others^36,47^. In DIPG preclinical models targeted by GD2 CAR T cells, Mount et al.^36^ reported peri-tumoral neuroinflammation during the acute phase of antitumor activity resulting in hydrocephalus, which was lethal in a fraction of animals. Richman et al.^47^ reported an enhanced antitumor activity of high-affinity anti-GD2 CAR constructs against human GD2-positive neuroblastoma xenografts while observing lethal central nervous system (CNS) toxicity comprising extensive brain CAR T-cell infiltration and proliferation as well as neuronal destruction.

Both CAR T approaches mentioned above are based on 14G2a clones that substantially differ from our scFv-mouse IgM-based construct. In the past CAR T generated from different Ig clones, one IgM- and two IgG-derived, were also associated with differences in terms of efficacy and side effects^16^. While comparisons with these models may be difficult to implement due to several variables (i.e., CAR structure, CAR transduction levels, gene engineering methods, and in vivo study design), again we confirm a reliable safety profile of the current second-generation anti-GD2 CAR. However, we are aware that the safety of the anti-GD2 CAR T may be confounded by lack of cross-reactivity with similar murine GD2, thus the safety of our anti-GD2 CAR T shall be carefully determined preclinically before translation into clinical trials.

To conclude, our in vivo data suggest the importance of CAR T cell homing in glioblastoma and tumor accessibility, as important factors in planning both preclinical and clinical phases for anti-glioblastoma cell-therapy approaches. At the same time, the study reveals the potential of locally injected cells to control glioblastoma proliferation, prompting further investigation aimed at this gene therapy strategy.

## Methods

### Glioblastoma cell culture and transduction

The study was approved by the Ethical and Institutional Review Board at the University Hospital of Modena (Prot. N.3600/C.E. September 2017) and was carried out in accordance with under relevant guidelines and regulations. Primary tumor samples and peripheral blood were obtained after signed informed consent from patients who underwent surgery at the Neurosurgery Unit (NOCSAE, Baggiovara, University Hospital of Modena). Tissue samples were collected during the 2017–2019 period. High-grade diagnosed glioma samples were dissociated into single cells with the Human Tumor Dissociation Kit (MACS, Myltenyi Biotec, Bergisch Gladbach, Germany; #130-095-929) using gentleMACS Octo Dissociator with heater protocols (Myltenyi Biotec) and plated in glioblastoma stem cell media (GSC). GSC media is composed of DMEM-F12 (Gibco, Sigma-Aldrich, Saint Louis, MO, USA; #21331-020/046) supplemented with B27 1× (ThermoFisher Scientific, MA, USA; #12587010), EGF (20 ng/mL, PeproTech, London, UK; #AF-100-15), bFGF (20 ng/mL, PeproTech; #AF-100-18B), 1% glutamine (EuroClone, Pero, MI, Italy; #ECB3000D), and 1% penicillin–streptomycin (CarloErba, Milano, Italy; #FA30WL0022100), while commercially available glioblastoma cell lines (T98G ATCC® CRL-1690^TM^ and A172 ATCC® CRL-1620, Manassas, Virginia, USA) were maintained in DMEM-F12 with 10% FBS (Carlo Erba; #FA30WS181B500), 1% glutamine (EuroClone), and 1% penicillin–streptomycin (CarloErba). Once cells started to form spheroids, the medium was replaced every 2–3 days by letting them settle to the bottom by gravity at 37 °C. The cell passage was performed every 3–5 days to prevent spheroids from growing too large and reseeding at a density of 100,000 cells/mL. For samples growing as a monolayer, the cell passage was performed every 3 days, and the reseeding density was 20,000 cells/cm^2^. Selected primary tumor samples (C3, C6, C10, and C12) and cell lines (T98G and A172) were transduced either by viral particles encoding for dsRED (retrovirus production was performed by the FLYRD18 packaging cell lines, as published^48^) or luciferase protein (Lentiviral particles for Firefly Luciferase by GeneCopoeia, MD, USA; #LPP-hLUC-Lv206-025-C), for either cell tracking in co-culture with CAR T cells or in in vivo studies. Primary GBM cells and GBM cell lines were routinely tested to evaluate antigen expression by flow cytometry and screened for the absence of mycoplasma (MycoAlert Mycoplasma Detection Kit and MycoAlert Control Set, Lonza, Basel, Switzerland; #LOLT07318, #LOLT07518). Authentication of T98G and A172 cell lines has been performed by the Leibniz Institute DSMZ–German Collection of Microorganisms and Cell Cultures GmbH, Braunschweig, Germany.

### GD2 expression in primary glioblastoma cells

The expression of GD2 antigen on isolated tumor cells was assessed by FACS analysis. Primary unconjugated mouse antihuman GD2 (BD, Franklin Lakes NJ, USA; #554272, 1:20) and secondary APC-conjugated goat antimouse Ig (APC Goat Anti-Mouse Ig polyclonal multiple adsorptions; BD; #550826, 1:20) were used for staining. All samples were acquired by BD FACSAria III (BD) and analyzed using BD FACSDiva software (BD).

### CAR T cells manufacturing and transduction

PBMCs were isolated from healthy donors or GBM affected patients (as approved by the Institutional Review Board), separated by a density gradient (Lymphoprep; Alere Technologies AS, Oslo, Norway; #1114545) and then plated in RPMI 1640 (Gibco; #52400-017) with 1% FBS, 1% glutamine, and 1% penicillin–streptomycin. Nonadherent cells were collected and prestimulated for 48 h in RPMI 1640 supplemented with 10% heat-inactivated defined FBS (HyClone Laboratories, UT, USA; #SH30070.03), 500 UI/mL rhInterleukin-2 (rhIL-2, Proleukin, Clinigen Healthcare Ltd, Stafforshire DE14 2WW, UK; #801313AY), and 7 μg/mL Phytohemagglutinin (PHA-M, Sigma-Aldrich; #L8629) at the concentration of 1 × 10^6^ cells/mL. Isolated T cell population underwent retroviral transductions to express a second-generation anti-GD2 CAR; for details refer to Prapa et al.^16^, and GFP control vector.

### Viability fluorescence assay

GD2 CAR T or GFP T cells were used as effectors (E) and human GD2-positive either primary glioblastoma cells or glioblastoma cell lines as targets (T), previously transduced with dsRED retroviral vectors. Cell viability of target cells at different effector-to-target ratios (E:T) was evaluated in 96-well black plates (Corning, Kennebuck, ME, USA; #3904) using GloMax Discover Multimode Microplate Reader (Promega, Madison, WI, USA). Cultures containing the medium alone or 1% Triton X-100 were used as controls, representing 100% and 0% cell viability, respectively. Average viability was calculated as 100 × (experimental fluorescence − 0% viability fluorescence)/(100% viability fluorescence − 0% viability fluorescence). Longtime point cell viability of target cells was assessed by mean fluorescence intensity (MFI) in FACS analysis. Representative fluorescence images of co-cultures over 96 h were collected by EVOS FL auto (Thermo Fisher Scientific).

### Luminex cytokine release assay

Forty-eight hours and 7 days co-culture supernatant levels of granzyme B, IFNγ, MIP-1α, TRAIL, TNFα, sFAS-ligand, CD137, TGF β1, GM-CSF, and IL-6 were measured with human custom ProcartaPlex kit (Thermo Fisher Scientific; #PPX-09-MXWCWUF, #PPX-10MXU629H, #EPX010-10420-901, # EXP01A-10249-901) by Luminex technology.

### In vitro 3D assays

In vitro 3D cultures were performed using two different approaches. dsRED-positive glioblastoma spheres were obtained by seeding 20,000 cells/well in 96-well plate used to reach sphere formation (Sphera Low-Attachment Surface, ThermoFisher; #174927). After 24 h glioblastoma spheres were co-cultured at 2:1 (E:T ratio) over 5 days assay. In addition, a 3D bioreactor (VITVO, Rigenerand, Modena, Italy; #F000001 (ref. ^49^)) was used to monitor the antitumor effect on target cells mediated by CAR T cells at an E:T ratio of 5:1 from 72 h to 5 days. The bioreactor was at first primed with media to ensure the complete wetting of the 3D matrix, then 5.6 × 10^5^ glioblastoma plain cells in 1.4 mL of culture media were injected into VITVO by a 3 mL syringe (Becton Dickinson and Co, Franklin Lakes, NJ, USA). After 24 h, effector T cells were added. Representative fluorescence images, including viability/cytotoxicity rate with LIVE (CalceinAM)/DEAD (EtBr) Kit (Life Technologies, California, USA; #L3224), were collected by EVOS FL auto.

### In vivo orthotopic glioblastoma models

Two delivery routes were investigated in animal experiments following the protocol approved by the national Ministry of Health and by the local Institutional Animal Care and Use Committee (Prot. N.706/2019-PR October 2019). To assess in vivo GD2 CAR T cells antitumor activity, an orthotopic model was generated in NOD/SCID mice (NOD. C. B-17-Prkdc^scid^/J, Charles River Laboratories Italia Srl, Lecco, Italy) similar to what was reported^50^. Eight- to 10-week-old mice were anesthetized with an intraperitoneal injection of Ketamine 150 mg/kg (Lobotor, Acme S.r.l. RE, Italy) and Xylazine 20 mg/kg (Sedaxylan, Dechra Veterinary Products Srl, Torino, Italy) mix solution. The head was shaved, and mice were immobilized in a stereotaxic frame apparatus (Just for mouse stereotaxic instrument Stoelting®, Dublin, Ireland), then scrubbed with 1% povidone-iodine solution. A 10 mm skin incision was made along the midline. The tip of a 27G needle mounted on a Hamilton syringe (Hamilton, Reno, NV, USA; #HAMI7803-07) served as the reference point. A 1 mm burr-hole was drilled into the skull 1 mm anterior and 2 mm to the right of the bregma. Firefly-luciferase-expressing patient-derived glioblastoma cells (C12.FFLuc;1 × 10^5^) were injected in 5 μL total volume, 3 mm deep to the bregma, corresponding to the center of the right caudate nucleus. The burr-hole was filled with bone wax (Surgical Specialties Corporation, Tijuana, Mexico; #901), and the incision was closed with metallic clips with the AutoClip System Kit (F.S.T, Heidelberg, Germany; # No. 12020-09 100). A subcutaneous injection of Ketoprofene 3 mg/kg (Ibifen- Istituto Biochimico Italiano, LT, Italy) was given for pain maintenance. For the intravenous delivery route, T cells were given via two doses of 1.5 × 10^6^ cells at day 8 and 1.9 × 10^6^ at day 20 or 5 × 10^6^ at day 7 and day 14, post intracranial tumor injection. For the in loco delivery route, tumor cells were injected either alone or co-injected with the effector T cells (GD2 CAR T or GFP T cells, E:T of 2:1 and 5:1) in 5 μL total volume. Animal models were monitored by bioluminescence. Isofluorane (Baxter, IL, USA) anesthetized animals were imaged using the IVIS Lumina XRMS Series III system (PerkinElmer, Waltham, MA, USA) 10–16 min after 150 mg/kg d-luciferin (Beetle Luciferin, Promega Co, Madison, USA; #E1605) was injected in each mouse subcutaneously. The photons emitted from the luciferase-expressing tumor cells were quantified using Living Image software (Caliper Life Sciences, Hopkinton, MA, USA). A pseudo-color image representing light intensity (blue = least intense and red=most intense) was generated and superimposed over the grayscale reference image. Animals were imaged once weekly beginning a week after injection. Mice were euthanized when they met euthanasia criteria (neurological deficits, 20–30% weight loss, signs of distress).

### Histology

Formalin-fixed, paraffin-embedded tumor specimens were cut and stained by hematoxylin-and-eosin staining (Sigma-Aldrich). For immunohistochemistry analysis, sections were retrieved in protease K (Sigma-Aldrich; #P2308) for 5 min at room temperature and incubated overnight at 4 °C with the primary anti-human mitochondria antibody at a dilution of 1:200 (MIT) (Millipore Corporation, Billerica, MA, USA; # MAB1273) to confirm human origin. Slides were then incubated with a biotinylated goat anti-mouse IgG (H + L) at a dilution of 1:200 (Vector Laboratories, Burlingame, CA; # BA9200) for 1 h at room temperature. Negative controls were run simultaneously omitting primary antibody while incubating with buffer. Staining was performed and visualized by 3.3 *O*-diaminobenzidine (DAB) (in brown, Vector Laboratories; #SK-4100). All slides were counterstained with Harris hematoxylin (Bio Optica, Milan, Italy). Sections were examined by Zeiss Axioskop (Zeiss, Oberkochen, Germany). Photomicrographs were acquired by an AxioCam ICc3 color camera and AxioVision software (Zeiss). Moreover, mouse brain tissue was placed in a pre-labeled base plastic mold partially filled with frozen tissue matrix (OCT), embedded completely in OCT compound, and then allowed to solidify at −20 °C. Frontal 8-µm-thick sections were cut and fixed in glutaraldehyde 2% and paraformaldehyde (PFA) 4% for 5 min in order to perform immunohistochemistry and immunofluorescence analyses, respectively, and stored in PBS at + 4 °C.

For immunohistochemistry analyses, frozen sections were blocked with 3% BSA in PBS for 30 min at room temperature and then incubated overnight at +4 °C with mouse anti-GD2 primary antibody (BD, #554272) diluted 1:100 in 1% BSA in PBS. After rinsing, slides were incubated for 1 h at room temperature with anti-mouse HRP-labeled secondary antibody (ThermoFisher, #31430) diluted 1:100. HRP was revealed by the DAB-based kit (Sigma Aldrich, #D3939-1). For immunofluorescence analyses frozen sections were incubated with mouse anti-GD2 (BD, #554272, 1:100) and rabbit anti-GFP (Abcam, #Ab290, 1:500) primary antibodies following the incubation with the secondary antibodies donkey anti-mouse Alexa546 (Life Technologies, #A10036, 1:200) and goat anti-rabbit Alexa488 (Life Technologies, #A11070, 1:200).

Nuclei were stained with 1 µg/ml 40,6-diamidino-2-phenylindole (DAPI) in PBS 1%. The multi-labeling immunofluorescence experiments were carried out avoiding cross-reactions between primary and secondary antibodies. Confocal imaging was performed using a Nikon A1 confocal laser scanning microscope. The confocal serial sections were processed with Fiji ImageJ software (NIH, Bethesda, MD, USA) in order to obtain 3D projections and image rendering was performed by Adobe Photoshop Software.

### Statistical analysis

Graph generation and statistical tests were conducted using GraphPad Prism version 6.0 (GraphPad Prism software, San Diego, CA, USA). In vitro data are expressed by means ± standard deviation (SD). Unpaired, two-tailed Student’s *t*-test was used to determine statistical significance. A level of *p* value <0.05 was used to designate significant differences. For survival analysis, data were analyzed using the Kaplan–Meier survival curves, and the log-rank test was used to measure differences among groups. Tumor bioluminescent signal expressed in total flux (p/s) over time was compared using a two-tailed *t*-test. Neither randomization nor blinding was done during the in vivo study.

## Supplementary information

Supplementary Information

## Data Availability

The datasets generated during and/or analyzed during the current study are available from the corresponding author on reasonable request.

## References

[1] McFaline-Figueroa JR, Lee EQ (2018). Brain tumors. Am. J. Med..

[2] Hanif F, Muzaffar K, Perveen K, Malhi SM, Simjee SU (2017). Glioblastoma multiforme: a review of its epidemiology and pathogenesis through clinical presentation and treatment. Asian Pac. J. Cancer Prev..

[3] Felthun J, Reddy R, McDonald KL (2018). How immunotherapies are targeting the glioblastoma immune environment. J. Clin. Neurosci..

[4] Weller M (2014). EANO guideline for the diagnosis and treatment of anaplastic gliomas and glioblastoma. Lancet Oncol..

[5] Migliorini D (2018). CAR T-cell therapies in glioblastoma: a first look. Clin. Cancer Res..

[6] Kalos M (2011). T cells with chimeric antigen receptors have potent antitumor effects and can establish memory in patients with advanced leukemia. Sci. Transl. Med..

[7] Salinas RD, Durgin JS, O’Rourke DM (2020). Potential of glioblastoma-targeted chimeric antigen receptor (CAR) T-cell therapy. CNS Drugs.

[8] Zimmermann K (2020). Design and characterization of an “all-in-one” lentiviral vector system combining constitutive Anti-GD2 CAR expression and inducible cytokines. Cancers.

[9] Majzner RG (2019). CAR T cells targeting B7-H3, a Pan-cancer antigen, demonstrate potent preclinical activity against pediatric solid tumors and brain tumors. Clin. Cancer Res..

[10] Traylor TD, Hogan EL (1980). Gangliosides of human cerebral astrocytomas. J. Neurochem..

[11] Mennel HD (1992). Expression of GD2-epitopes in human intracranial tumors and normal brain. Exp. Toxicol. Pathol..

[12] Mennel HD, Bosslet K, Geissel H, Bauer BL (2000). Immunohistochemically visualized localisation of gangliosides Glac2 (GD3) and Gtri2 (GD2) in cells of human intracranial tumors. Exp. Toxicol. Pathol..

[13] Longee DC (1991). Disialoganglioside GD2 in human neuroectodermal tumor cell lines and gliomas. Acta Neuropathol..

[14] Golinelli G (2020). Targeting GD2-positive glioblastoma by chimeric antigen receptor empowered mesenchymal progenitors. Cancer Gene Ther..

[15] Doronin II (2014). Ganglioside GD2 in reception and transduction of cell death signal in tumor cells. BMC Cancer.

[16] Prapa M (2015). A novel anti-GD2/4-1BB chimeric antigen receptor triggers neuroblastoma cell killing. Oncotarget.

[17] Majzner RG, Mackall CL (2019). Clinical lessons learned from the first leg of the CAR T cell journey. Nat. Med..

[18] Hou AJ, Chen LC, Chen YY (2021). Navigating CAR-T cells through the solid-tumour microenvironment. Nat. Rev. Drug Discov..

[19] Khan JF (2019). Application of CAR T cells for the treatment of solid tumors. Prog. Mol Biol Transl Sci.

[20] Maccalli C, Parmiani G, Ferrone S (2017). Immunomodulating and immunoresistance properties of cancer-initiating cells: implications for the clinical success of immunotherapy. Immunol. Invest..

[21] Colombo M (2018). Cancer cells exploit notch signaling to redefine a supportive cytokine milieu. Front. Immunol..

[22] Candini O (2020). Author correction: a novel 3D in vitro platform for pre-clinical investigations in drug testing, gene therapy, and immuno-oncology. Sci. Rep..

[23] Seano G, Jain RK (2020). Vessel co-option in glioblastoma: emerging insights and opportunities. Angiogenesis.

[24] Medikonda, R., Dunn, G., Rahman, M., Fecci, P. & Lim M. A review of glioblastoma immunotherapy. *J. Neurooncol.* **151**, 41–53 (2020). 10.1007/s11060-020-03448-1 32253714

[25] Murciano-Goroff YR, Warner AB, Wolchok JD (2020). The future of cancer immunotherapy: microenvironment-targeting combinations. Cell Res..

[26] Ahmed N (2010). HER2-specific T cells target primary glioblastoma stem cells and induce regression of autologous experimental tumors. Clin. Cancer Res..

[27] Kahlon KS (2004). Specific recognition and killing of glioblastoma multiforme by interleukin 13-Zetakine redirected cytolytic T cells. Cancer Res..

[28] Sengupta S, Mao G, Gokaslan ZS, Sampath P (2017). Chimeric antigen receptors for treatment of glioblastoma: a practical review of challenges and ways to overcome them. Cancer Gene Ther..

[29] O'Rourke DM (2017). A single dose of peripherally infused EGFRvIII-directed CAR T cells mediates antigen loss and induces adaptive resistance in patients with recurrent glioblastoma. Sci. Transl. Med..

[30] Goff SL (2019). Pilot trial of adoptive transfer of chimeric antigen receptor–transduced T cells targeting EGFRvIII in patients with glioblastoma. J. Immunother..

[31] Morgan RA (2012). Recognition of glioma stem cells by genetically modified T cells targeting EGFRvIII and development of adoptive cell therapy for glioma. Hum. Gene Ther..

[32] Ahmed N (2017). HER2-specific chimeric antigen receptor–modified virus-specific T cells for progressive glioblastoma: a phase 1 dose-escalation trial. JAMA Oncol..

[33] Brown CE (2015). Bioactivity and safety of IL13R 2-redirected chimeric antigen receptor CD8+ T cells in patients with recurrent glioblastoma. Clin. Cancer Res..

[34] Brown CE (2016). Regression of glioblastoma after chimeric antigen receptor T-cell therapy. N. Engl. J. Med..

[35] Choi BD, Maus MV, June CH, Sampson JH (2019). Immunotherapy for glioblastoma: adoptive T-cell strategies. Clin. Cancer Res..

[36] Mount CW (2018). Potent antitumor efficacy of anti-GD2 CAR T cells in H3-K27M+ diffuse midline gliomas. Nat. Med..

[37] Murty S (2020). Intravital imaging reveals synergistic effect of CAR T-cells and radiation therapy in a preclinical immunocompetent glioblastoma model. OncoImmunology.

[38] Heczey A (2020). Anti-GD2 CAR-NKT cells in patients with relapsed or refractory neuroblastoma: an interim analysis. Nat. Med..

[39] Seidel, S., Garvalov, B. K. & Acker, T. in *Stem Cell Protocols* (ed. Rich, I. N.) 263–275 (Springer New York, 2015). http://link.springer.com/10.1007/978-1-4939-1785-3_19.

[40] Benmebarek M-R (2019). Killing mechanisms of chimeric antigen receptor (CAR) T cells. Int J. Mol. Sci..

[41] Wong HY, Schwarz H (2020). CD137/CD137 ligand signalling regulates the immune balance: a potential target for novel immunotherapy of autoimmune diseases. J. Autoimmun..

[42] West, A., et al. The role of interleukin‑6‑STAT3 signalling in glioblastoma (Review). *Oncol. Lett.*10.3892/ol.2018.9227 (2018). 10.3892/ol.2018.9227 PMC6144698 30250528

[43] Jiang, Z. et al. IL-6 trans-signaling promotes the expansion and anti-tumor activity of CAR T cells. *Leukemia* **35**, 1380–1391 (2020). 10.1038/s41375-020-01085-1 33168950

[44] Filley AC, Henriquez M, Dey M (2018). CART immunotherapy: development, success, and translation to malignant gliomas and other solid tumors. Front. Oncol..

[45] Brown CE (2018). Optimization of IL13Ra2-targeted chimeric antigen receptor T cells for improved anti-tumor efficacy against glioblastoma. Mol. Ther..

[46] Wang D (2020). Chlorotoxin-directed CAR T cells for specific and effective targeting of glioblastoma. Sci. Transl. Med..

[47] Richman SA (2018). High-affinity GD2-specific CAR T cells induce fatal encephalitis in a preclinical neuroblastoma model. Cancer Immunol. Res..

[48] Marx JC (1999). High-efficiency transduction and long-term gene expression with a murine stem cell retroviral vector encoding the green fluorescent protein in human marrow stromal cells. Hum. Gene Ther..

[49] Candini O (2019). A novel 3D in vitro platform for pre-clinical investigations in drug testing, gene therapy, and immuno-oncology. Sci. Rep..

[50] Bianco J (2017). Novel model of orthotopic U-87 MG glioblastoma resection in athymic nude mice. J. Neurosci. Methods.

