# Supplementary material for: GD2 CAR T cells against human glioblastoma
Source: NPJ Precis Oncol. 2021 Oct 27;5:93. doi: 10.1038/s41698-021-00233-9 (PMC8551169; doi:10.1038/s41698-021-00233-9)
Supplement: Supplementary file 1 — Supplementary Information [file 41698_2021_233_MOESM1_ESM.pdf]

Supplementary figure 1

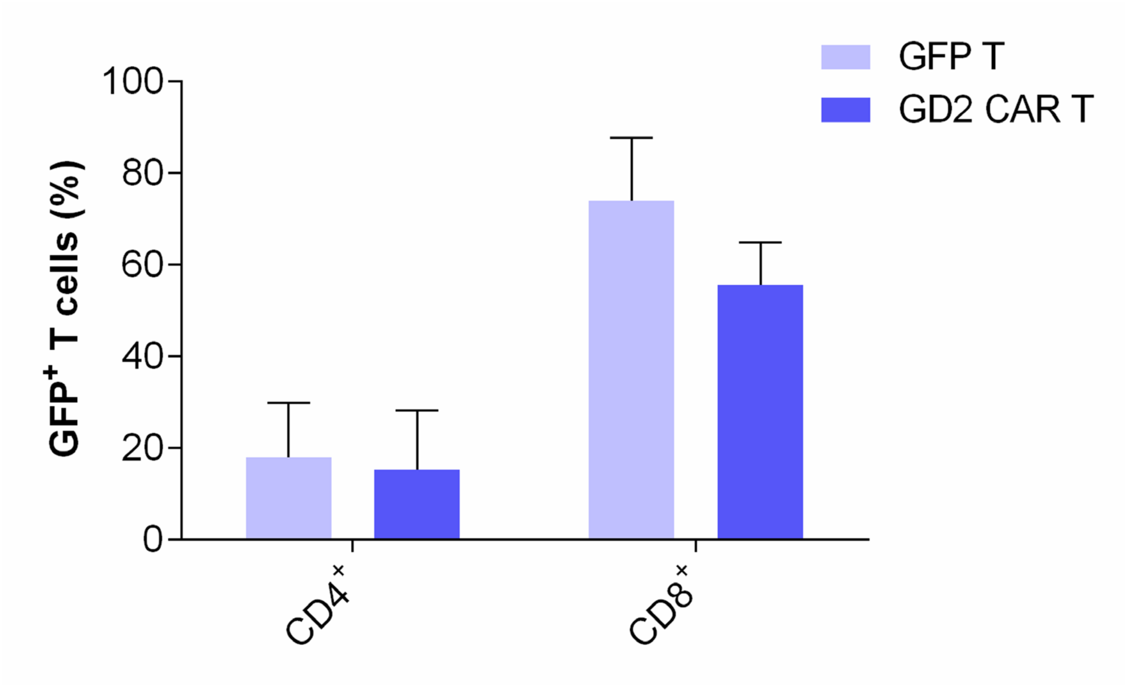

Supplementary Figure 1. Transduction efficiency. Detection of GFP$^+$ T cells within CD4$^+$ and CD8$^+$ subpopulations in GD2 CAR T (15,2±13,0% and 55,6±9,2%) and GFP T (17,9±12,0% and 74,0±13,6%) cells. *p value* not significant among groups by unpaired two-tailed t-test. Data are shown as mean ± SD from six PBMC donors.

Supplementary figure 2

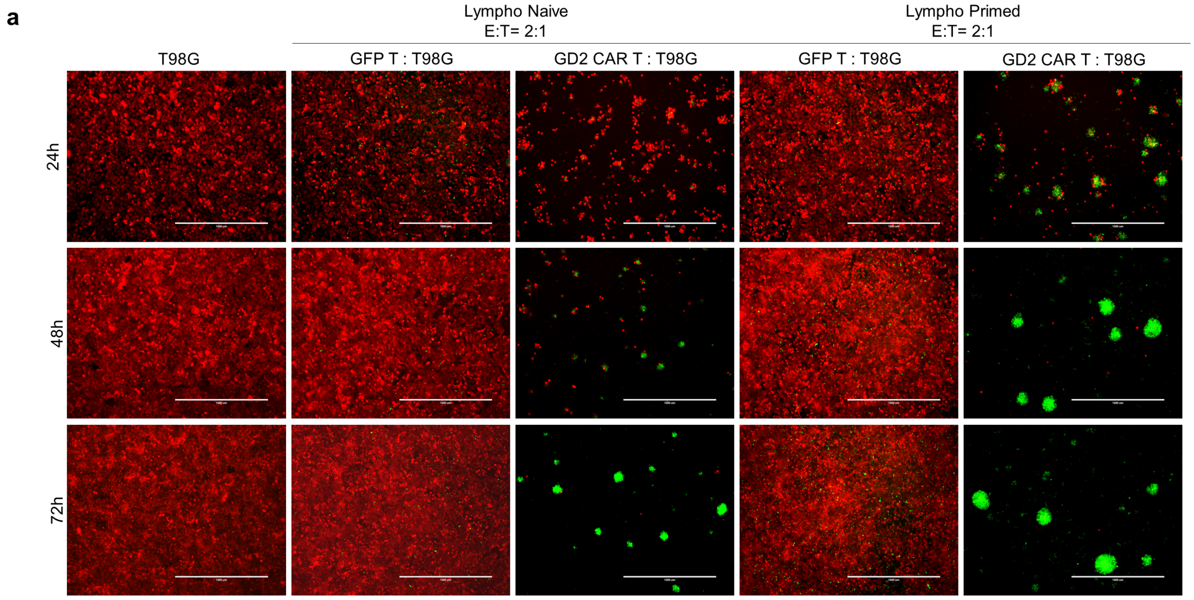

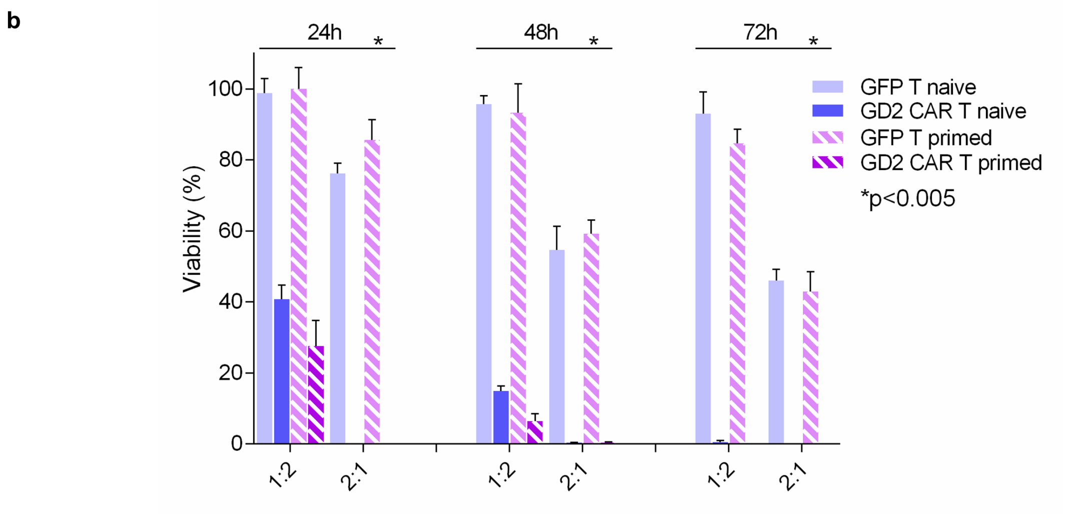

Supplementary Figure 2. Tumor-primed and naïve GD2 CAR T cells show robust activation in cluster and killing activity against T98G GD2^{high}. a, Representative fluorescence micrographs of dsRED T98G glioblastoma (red) cells in co-culture with GD2 CAR T (green) and GFP T control cells (green) at 2:1 E:T ratio. The scale bar: 1000 µm. **b,** After 24, 48, 72h tumor viability at 2:1 and 5:1 E:T ratios is calculated by fluorescence assay as reported in Materials and Methods. Data are shown as mean ± SD from three technical replicates; *p values* are calculated by unpaired two-tailed t-test.

Supplementary figure 3

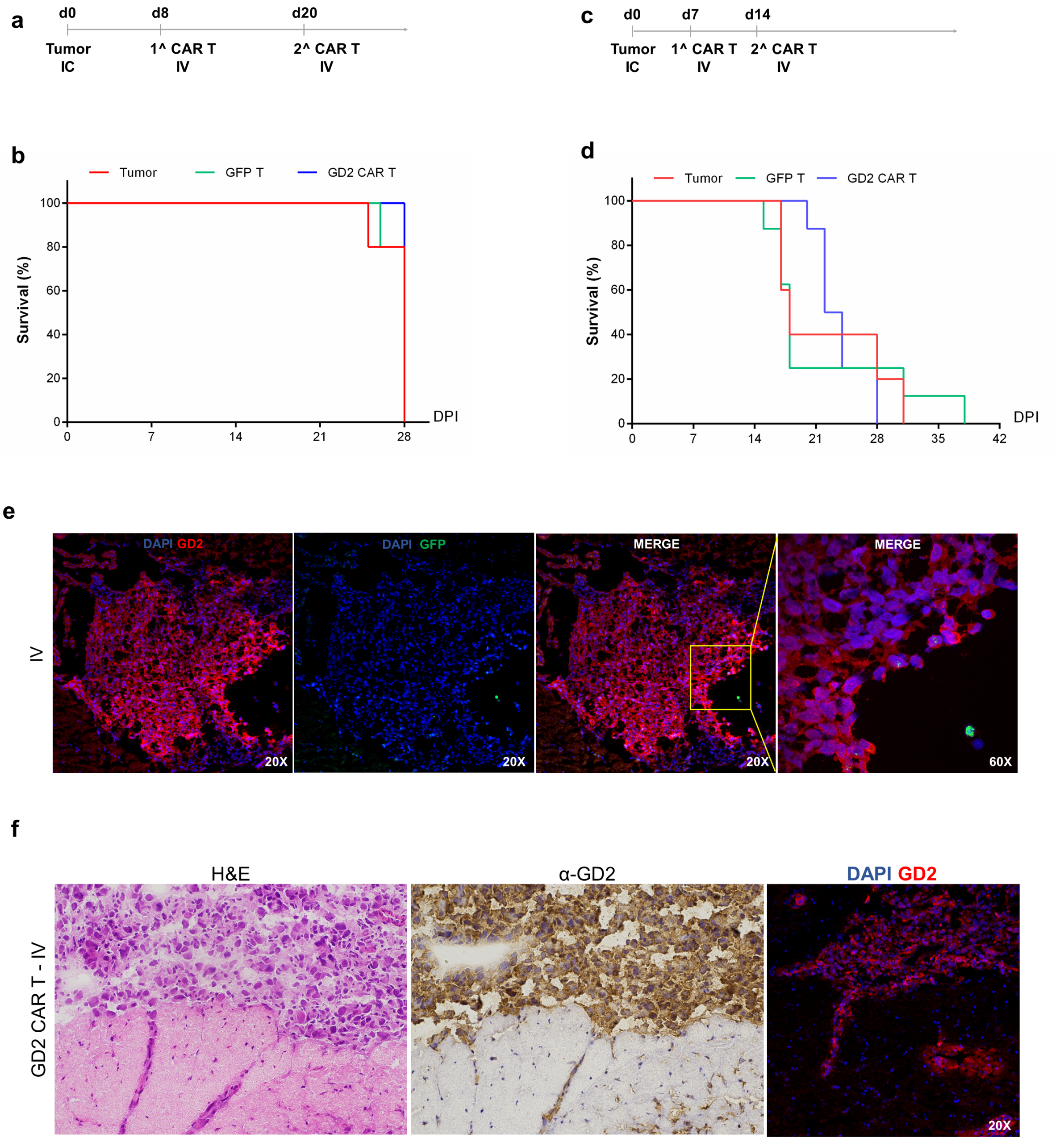

Supplementary Figure 3. IV administration of GD2 CAR T cell does not improve survival in the orthotopic glioblastoma model. a, Schematic outline of intravenous CAR T treatment. NOD/SCID mice (n=5) are inoculated intracranially with 1×10^5 C12.FFLuc GBM cells followed by intravenous injections of $1.5x10^6$ (1^ CAR T IV) at day 8 and $1.9x10^6$ (2^ CAR T IV) at day 20 with either GD2 CAR T or GFP control T cells. Control mice are injected with PBS. **b,** Kaplan-Meier survival curve. **c,** Schematic outline of intravenous CAR T treatment. NOD/SCID mice (n=5) are inoculated intracranially with 1×10^5 C12.FFLuc GBM cells followed by intravenous injections of $5x10^6$ at days 7 and 14 with either GD2 CAR T or GFP control T cells. Control mice are injected with PBS. **d,** Kaplan-Meier survival curve. **e,** Immunofluorescence representative micrographs showing the lack of GFP^+ infiltration in the explanted GBM treated IV and **f,** GD2 staining in explanted tumors treated IV show a significant and persisting antigen expression persistence by immunohistochemistry (middle column) and immunofluorescence (right column) stains.

Supplementary figure 4

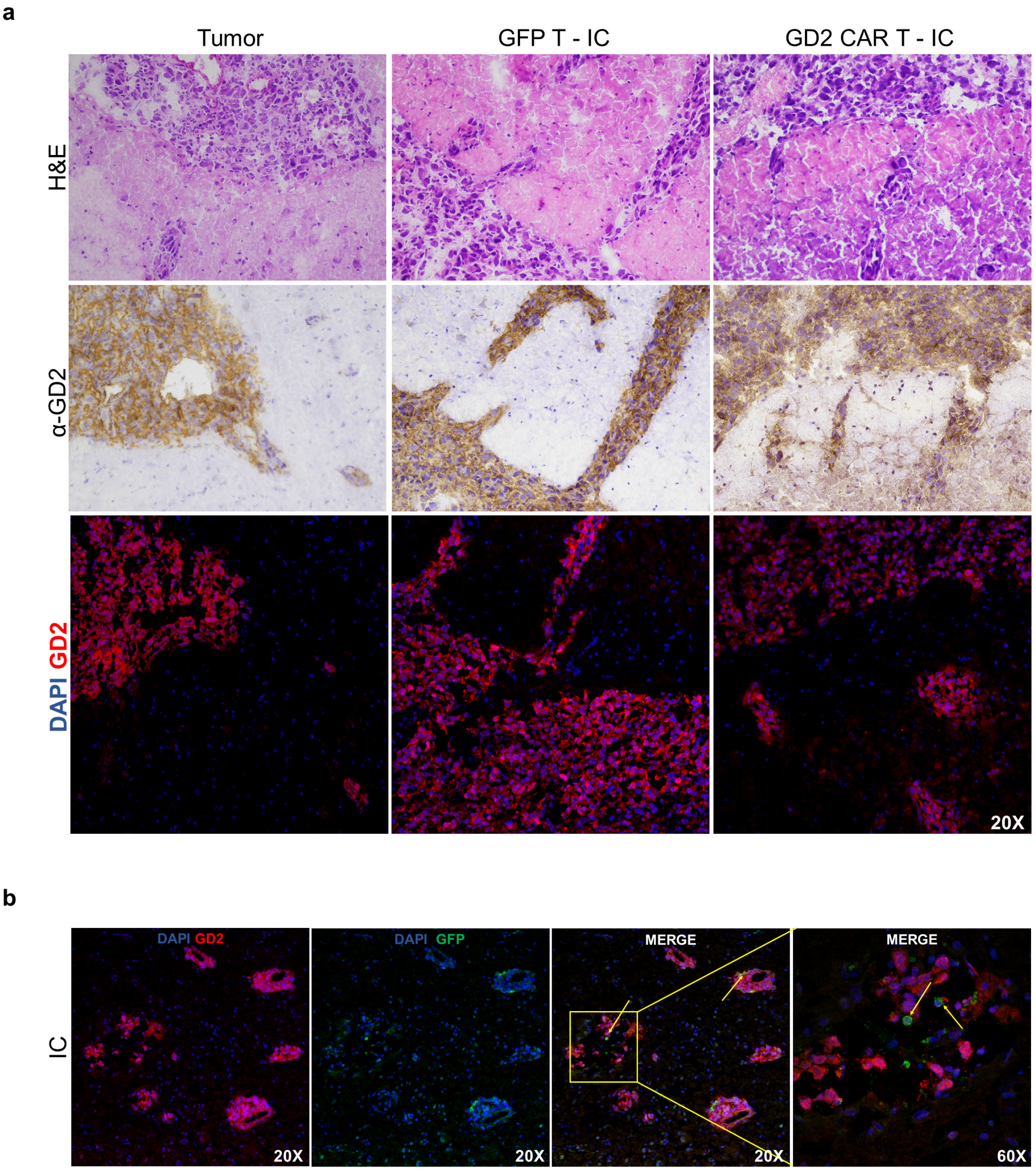

Supplementary Figure 4. GD2 antigen persistence and GFP⁺ T elements in explanted tumors treated IC. a, GD2 is extensively expressed in explanted orthotopic models showing a negligible loss of antigen by both immunohistochemistry (middle row) and immunofluorescence (bottom row) stains. **b,** Immunofluorescence representative micrographs showing the persistence of GFP⁺ T cells in the explanted GBM treated by IC injection.
